# Association Between Substantia Nigra Hyperechogenicity and Central Macular Thickness in Parkinson’s Disease

**DOI:** 10.3390/biomedicines14071600

**Published:** 2026-07-17

**Authors:** Marko Svetel, Dragan Spaić, Milija Mijajlović, Gorica Marić, Marija Božić, Jelena Vasilijević, Ana Dimitrijević, Vladimir Milutinović, Nada Avram, Marina Svetel

**Affiliations:** 1 Clinic for Eye Diseases, University Clinical Center of Serbia, 11000 Belgrade, Serbia; markosvetel.mfub@gmail.com (M.S.); marija.bozic@med.bg.ac.rs (M.B.); bkjelena@gmail.com (J.V.); ana.m.dimitrijevic@gmail.com (A.D.); dr.vladimir.milutinovic@gmail.com (V.M.); 2 Department of Primary Health Care and Public Health, Faculty of Medicine Foca, University of East Sarajevo, 73300 Foca, Bosnia and Herzegovina; 3 Clinic for Neurology, University Clinical Center of Serbia, 11000 Belgrade, Serbia; milijamijajlovic@yahoo.com (M.M.); marinasvetel@gmail.com (M.S.); 4 Faculty of Medicine, University of Belgrade, 11000 Belgrade, Serbia; 5 Institute of Epidemiology, Faculty of Medicine, University of Belgrade, 11000 Belgrade, Serbia; gorica.maric@med.bg.ac.rs; 6 Department of Ophthalmology, Faculty of Medicine Foca, University of East Sarajevo, 73300 Foca, Bosnia and Herzegovina; nadaavram@gmail.com

**Keywords:** Parkinson’s disease, central macular thickness, substantia nigra hyperechogenicity, optical coherence tomography (OCT), transcranial sonography (TCS), biomarkers, retinal thinning

## Abstract

**Background/Objectives:** This study investigates the correlation between substantia nigra (SN) hyperechogenicity, detected via transcranial sonography (TCS), and retinal thinning in patients with Parkinson’s disease (PD). **Methods:** In this cross-sectional study, 86 PD patients (172 eyes) underwent clinical assessment (UPDRS, Hoehn–Yahr), TCS for SN area measurement, and optical coherence tomography (OCT) for retinal nerve fiber layer (RNFL), ganglion cell-inner plexiform layer (GCIPL), and macular thickness analysis. **Results:** PD patients exhibited significant thinning of the GCIPL, average RNFL, and central macular thickness compared to healthy controls (*p* < 0.001 in all instances). Pathological SN hyperechogenicity (>0.19 cm^2^) was detected in 85.9% of patients with a preserved temporal bone window (71 out of 86). A significant negative correlation was found specifically between the SN echogenic area and central macular thickness (r = −0.248, *p* = 0.003). Patients with pathological SN findings had a significantly thinner central macula (*p* = 0.006) compared to those with a normal SN, while no significant correlations were observed between the SN area and RNFL or GCIPL. **Conclusions:** These findings demonstrate a specific correlation between SN hyperechogenicity and central macular thinning. While RNFL and GCIPL effectively differentiate PD from controls, the central macula might serve as a promising biomarker candidate reflecting SN changes. Further longitudinal studies are required to evaluate the role of these combined markers in the PD diagnostic algorithm.

## 1. Introduction

For decades, Parkinson’s disease (PD) was clinically defined as a localized motor disorder caused by the loss of dopamine-producing neurons in the substantia nigra (SN). However, PD is not a simple neurotransmitter deficiency, but a complex, multi-system, neurodegenerative process.

Pathologically, the core hallmark of PD is the widespread accumulation of misfolded α-synuclein proteins. These toxic proteins aggregate inside surviving neurons to form Lewy bodies and Lewy neurites, disrupting essential intracellular transport mechanisms. The precise molecular cascade driving this neurodegeneration is highly complex and multifactorial. Key mechanisms include chronic mitochondrial dysfunction, which leads to a severe energy crisis in cells. This state triggers the excessive production of reactive oxygen species and profound oxidative stress [1]. Additionally, cells suffer from impaired proteostasis, failing to clear damaged proteins [2]. This failure involves both the ubiquitin–proteasome system and defective autophagy–lysosome clearance pathways. Concurrently, a persistent neuroinflammatory response is driven by the overactivation of resident microglia cells. These activated microglia release pro-inflammatory cytokines that further accelerate the ongoing neuronal death.

Furthermore, patients with PD exhibit abnormal iron deposition in the brain [3]. Pathological iron deposition can induce α-synuclein accumulation, over expression, and abnormal folding, leading to the generation of Lewy bodies and giving rise to PD. Zhao et al. (2023) and Acosta-Cabronnero et al. (2017) showed that patients with PD had significantly higher iron deposition in the SN than the control group, and the abnormal region of iron deposition in the brain coincided with the region of ɑ-synuclein distribution [4,5]. Excessive iron accumulation is directly associated with oxidative stress and neuronal damage, and this oxidative stress is thought to contribute significantly to the degeneration of dopaminergic neurons observed in PD [6].

The diagnosis of PD primarily relies on the presence of cardinal motor symptoms [7]. However, there is an urgent clinical need for reliable biomarkers to facilitate early identification, monitor disease progression, and differentiate PD from atypical parkinsonian syndromes. Despite significant advancements in identifying PD through genetic, biochemical, and functional neuroimaging tests, there remains a need for more accessible and precise biomarkers with sufficient specificity for accurate diagnosis and monitoring of disease evolution.

Transcranial parenchymal echosonography (TCS) has emerged as a valuable diagnostic instrument, although it is not an independent diagnostic test for PD. Both the European Federation of Neurological Societies and the Movement Disorder Society have acknowledged the diagnostic utility of TCS, not only for differentiating PD from atypical and secondary parkinsonian disorders but also for early diagnosis and the detection of subjects at risk for PD (Level A recommendation) [8]. However, TCS findings should be interpreted with caution in cases of atypical parkinsonism due to subtype differences and overlapping clinical features [9]. This technology is non-invasive, cost-effective, and highly reproducible, serving as an excellent complement to clinical assessment and more costly modalities such as Magnetic Resonance Imaging (MRI) or Single-Photon Emission Computed Tomography (SPECT). Approximately 90% of individuals with idiopathic PD exhibit a distinctive finding on TCS: SN hyperechogenicity beyond the pathological threshold [10,11].

TCS’s sensitivity in the diagnosis of PD is between 91 and 100% [12]. A research investigation regarding the diagnostic significance of PD conducted by Tao et al. (2019) showed that the sensitivity and specificity of SN hyperechogenicity for PD were 84 and 85%, respectively, the sensitivity and specificity for distinguishing PD from healthy people were 85 and 89%, respectively, and the sensitivity and specificity to distinguish PD from other Parkinsonian syndromes were 82 and 74%, respectively [13].

Concurrently, numerous visual abnormalities, such as alterations in visual acuity, contrast sensitivity, color vision, and compromised eye movements, are detected in up to 92% of PD patients [14]. Optical Coherence Tomography (OCT) was first employed over twenty years ago as a non-invasive method to examine the retinal anatomy responsible for these deficits [15]. Recent literature consistently demonstrates that retinal thinning, particularly affecting macular areas, can differentiate PD patients from healthy controls [16,17,18,19,20]. Given that retinal thinning is prevalent in PD and that TCS serves as a key complementary neuroimaging technique, we intend to investigate the direct correlation between SN hyperechogenicity and specific retinal structures. To the best of our knowledge, the relationship between these two imaging modalities and potential biomarkers remains insufficiently explored.

## 2. Materials and Methods

Our study was observational and cross-sectional. We recruited our cohort at the Department of Neurodegenerative Diseases, Clinic for Neurology, University Clinical Center of Serbia (UCCS), from 1 June 2022 to 31 December 2024. Following neurological assessment, patients were referred to the Clinic of Ophthalmology UCCS for further evaluation.

The study was approved by the Ethics Committee of the Faculty of Medicine, University of Belgrade (No. 25/III-18) and the Ethics Committee of the University Clinical Center of Serbia (No. 1600/13). The study was conducted in accordance with the principles of the Declaration of Helsinki.

Informed consent was acquired from all patients involved in the study following a comprehensive explanation of the research.

### 2.1. Participants

The cohort included 86 patients (172 eyes) diagnosed with PD according to the Movement Disorder Society (MDS) clinical diagnostic criteria [7]. All participants were examined by a neurologist specializing in movement disorders.

Eighty-six volunteers (172 eyes) attending the Clinic of Ophthalmology for routine visual acuity assessments were selected as healthy controls. None of them had a history of ocular problems. Only minor refractive errors, incipient cataracts, and dry eyes were allowed. None of the healthy controls suffered from neurodegenerative diseases. Each PD patient was matched with a healthy control partner based on sex and age.

### 2.2. Clinical Assessment

Clinical and demographic data, including current age, sex, age at onset, disease duration, and levodopa daily dose were collected for all PD patients. Disease severity was evaluated using the Hoehn–Yahr scale [21] and the MDS-Unified Parkinson’s Disease Rating Scale (MDS-UPDRS) [22]. Patients did not discontinue their dopaminergic medication before the examination.

A complete ophthalmologic evaluation was performed on all PD patients and control subjects, incorporating best-corrected visual acuity (Snellen), intraocular pressure measurement (Goldmann applanation tonometry), slit-lamp biomicroscopy, gonioscopy, fundus examination via indirect ophthalmoscopy, and OCT.

### 2.3. Criteria for Exclusion

Exclusion criteria for PD patients and healthy controls were as follows: a history of glaucoma, suspicious optic nerve cupping, myopia exceeding −6 diopters, hyperopia of +6 diopters or greater, or any other ocular or systemic disorder capable of modifying the retina or impacting OCT analysis (diabetes mellitus, hypertension, systemic lupus erythematosus, etc.). Additionally, patients diagnosed with other neurological diseases (e.g., multiple sclerosis or Alzheimer’s disease), those who underwent intraocular surgery, or those who experienced ocular trauma were excluded from the study.

### 2.4. Optical Coherence Tomography Assessment

Retinal thickness measurements in PD patients and healthy controls were conducted using the SD-OCT device (RTVue-100, Optovue, Fremont, CA, USA). Values (expressed in µm) were obtained for the peripapillary retinal nerve fiber layer (RNFL), as well as the outer and inner macular layers.

In accordance with several other publications on this topic [16,23], results for each eye were acquired separately, and values recorded from each eye were included in the statistical analysis.

RNFL thickness was evaluated using a distinct scanning pattern: the area scanned was 4.9 mm centered on the optic disc, consisting of 13 concentric circles with diameters ranging from 1.3 mm to 4.9 mm (at 0.3 mm intervals) and 12 radial lines. The total number of data points was 14,241 with the scan time of 0.55 s.

The macular retinal thickness was measured in nine segments following the Early Treatment Diabetic Retinopathy Study (ETDRS) grid. The fovea center has a diameter of 1 mm, the parafovea ranges from 1 mm to 3 mm in diameter, and the perifovea spans from 3 mm to 5 mm in dimension. Both the parafovea and perifovea were individually segmented into four quadrants: superior, inferior, nasal, and temporal.

Multiple scans were conducted, and a quality rating of 40 or higher was required for analysis. The same observer evaluated all OCT images. The OCT grader and the TCS examiner were masked to clinical data and to each other’s measurements. The primary independent variables included the ganglion cell-inner plexiform layer complex (GCIPL) thickness, average RNFL thickness, and total macular thickness assessed over nine segments (central macula, parafoveal, and perifoveal areas).

### 2.5. Transcranial Parenchymal Ultrasonography (TCS)

TCS was conducted at the University Clinical Center of Serbia, specifically within the Clinic for Neurology’s Department of Ultrasound Diagnostics. Data acquisition was performed using a high-resolution FUJIFILM ARIETTA 650 DI system (Fujifilm, Tokyo, Japan) equipped with a 2–3.5 MHz phased-array transducer. All examinations adhered to a standardized clinical protocol, utilizing established ultrasound landmarks to identify specific insonation planes.

The assessment employed a transtemporal approach, with the probe positioned along the orbito-meatal line at the anterior, middle, or posterior temporal acoustic windows. This axial scanning methodology leverages regions of minimal cortical bone thickness to optimize ultrasound wave penetration. Within the ipsilateral field, the SN, red nucleus, and raphe nuclei were identified by their hyperechoic properties.

For precise quantification, B-mode images were subjected to a 3- to 4-fold magnification. The hyperechoic region of the SN was assessed by manually encircling the signal along its outer boundary to calculate the total area in square centimeters (cm^2^). Based on the current numeric criteria for our ultrasound laboratory and the ultrasound system applied, an area of <0.19 cm^2^ was interpreted as normal echogenicity, while values greater than 0.19 cm^2^ were defined as pathological SN hyperechogenicity (with values ranging from 0.19 to 0.24 cm^2^ classified as moderate hyperechogenicity, and areas exceeding 0.24 cm^2^ designated as marked hyperechogenicity).

### 2.6. Statistical Evaluation

Descriptive and inferential statistics were employed for data analysis. Numerical data were expressed as mean ± standard deviation, while nominal and ordinal data were expressed as absolute numbers and proportions. Group differences were assessed using Student’s *t*-test and the chi-square test. The association between relevant parameters was evaluated using Pearson and Spearman correlation coefficients.

In order to further evaluate the relationship between central macular thickness and the SN echogenic area, we performed a multivariate analysis with the inclusion of the following potential confounders: age, sex, disease duration, UPDRS score, HY stage and LED (levodopa).

All analyses were performed in SPSS version 20, with a *p*-value below 0.05 considered statistically significant.

## 3. Results

We analyzed 49 (57%) males and 37 (43%) females. The mean actual age was 59.2 ± 11.9 years, with a mean age at disease onset of 52.1 ± 12.6 years and a mean disease duration of 6.7 ± 5.6 years. The cohort exhibited a total UPDRS score of 40.7 ± 22.5. According to the Hoehn and Yahr scale, most patients were at stage II (40.7%), followed by stage I (23.3%) and stage III (15.2%).

When comparing retinal thickness between PD patients and the healthy control (HC) group, the GCIPL, average RNFL, and central macular thickness (M1) were statistically significantly thinner in the PD group. Furthermore, the inner (superior, inferior, temporal) and outer (superior, inferior, temporal) macular segments were also statistically significantly reduced in PD patients (Table 1).

Regarding TCS findings, 55 patients (64%) exhibited bilateral pathological SN hyperechogenicity, while 16 patients (18.6%) showed no bilateral enlargement. Unilateral or bilateral pathological findings were detected in a total of 61 patients (85.9%), whereas 10 patients (14.1%) had normal findings on both sides. Data were missing for 15 patients (17.4%) due to an inadequate temporal bone window.

When comparing patients with pathological SN hyperechogenicity (area > 0.19 cm^2^) to those with normal SN area (≤0.19 cm^2^), the only statistically significant difference observed was in the central macular thickness (M1) (*p* = 0.006). In cases of pathological SN findings, the M1 segment was significantly thinner. No significant differences were found for GCIPL (*p* = 0.219), average RNFL (*p* = 0.636), or other macular segments (Table 2).

Finally, a significant negative correlation was established specifically between the SN echogenic area and the thickness of the central macula (r = −0.248, *p* = 0.003).

The results of the multivariate analysis demonstrated that the relationship between central macular thickness and the SN echogenic area remained statistically significant even after controlling for potential confounders (*p* = 0.007). None of the additionally included variables emerged as significant confounding factors: age (*p* = 0.085), sex (*p* = 0.064), disease duration (*p* = 0.177), UPDRS score (*p* = 0.689), HY stage (*p* = 0.682), and levodopa daily dose (*p* = 0.723).

## 4. Discussion

This study investigates the correlation between SN hyperechogenicity and retinal thinning in PD patients, aiming to fill a gap in the literature regarding the intersection of these two diagnostic indicators.

In the first part of our study, our results (previously published in [24,25]) confirmed that the GCIPL, average RNFL, and central macular thickness, along with most other macular segments, were significantly thinner in PD patients compared to healthy controls. These findings are in accordance with the recent literature [16,17,18,20] suggesting that these segments effectively differentiate parkinsonian patients from the healthy population.

Retinal thickness is significantly influenced by a variety of physiological, anatomical, systemic, and technical factors. Understanding these variables is critical for the accurate interpretation of OCT scans in clinical practice. Consequently, in our investigation, the control group was age- and sex-matched and additionally, all control subjects with potential diseases that may influence retinal thickness were excluded from the study.

Although the precise mechanisms underlying retinal thinning remain to be fully elucidated, post-mortem evidence indicates a significant decrease in retinal dopamine levels among PD patients [26]. This neurochemical deficit is directly substantiated by structural tissue alterations at the cellular level. Ortuño-Lizarán et al. (2020) demonstrated a profound post-mortem loss of dopaminergic amacrine cells in PD retinas, ranging from 26% to 58%, with a unique topographic preference for the inferotemporal sector of the macula [27]. This loss further disrupts the essential communication between amacrine cells and retinal ganglion cells, ultimately leading to the atrophy of the ganglion cell layer and its associated nerve fibers [17]. Additionally, the presence of α-synuclein aggregates, a pathological signature of PD, has been identified within various retinal layers, specifically affecting dopaminergic amacrine cells in humans [27,28], as well as in transgenic mice [29], ultimately resulting in visual dysfunctions.

The correlation between reduced retinal thickness and nigral dopaminergic loss was first established by Ahn et al. [30] using dopamine transporter PET imaging. By showing that early macular thinning, predominantly localized to the inferior and temporal sectors, significantly correlates with central dopaminergic depletion, Ahn et al. (2018) [30] provided a vital clinical link. This topographically specific in vivo OCT thinning closely parallels the localized, intraretinal dopaminergic cell loss proven histopathologically by Ortuño-Lizarán et al. (2020) [27], validating that non-invasive imaging markers reflect true underlying cellular degradation.

Mounting evidence proposes that PD pathology may originate outside the central nervous system, spreading from peripheral sites like the autonomic or enteric nervous systems. In this context, the retina is of particular interest as an embryological extension of the diencephalon, sharing neuroectodermal origins and dopaminergic similarities with the brain [31,32]. These features position the retina as an ideal substrate for exploring early neurodegenerative processes in PD [33,34]. Interestingly, phosphorylated α-synuclein has been detected concurrently in the brain and retina even during premotor stages [28,35].

In accordance with this finding, Wagner et al. [20] revealed that in their prospective monitoring of numerous healthy individuals, among whom 0.1% developed PD, retinal layer thinning was also noticed prior to the onset of motor symptoms. These findings pointed out that retinal thickness might be a prodromal or premotor biomarker that could identify PD before a significant degree of damage has occurred and at a stage when neuroprotective therapies could halt or slow neuronal loss. Although retinal thinning was demonstrated in numerous studies in large cohorts of PD patients and these findings should not be neglected, the clinical application of these results and acceptance of retinal thickness as a biomarker is far away from routine practice and recommendations.

In the second part of our study, we focused on the relationship between these retinal changes and TCS findings. We detected pathological SN hyperechogenicity in 85.9% of our patients. Given that TCS is acknowledged by the EFNS and MDS as a tool for early diagnosis and identifying subjects at risk (Level A), we aimed to see if retinal thickness mirrors these findings. Interestingly, patients with pathological SN echogenicity differed from those with normal SN only in central macular thickness (M1).

The mechanism by which SN hyperechogenicity on TCS develops in patients with PD remains poorly understood. Iron ion deposition, reductions in neuromelanin, and glial cell hyperplasia are the primary areas of attention in the studies that are currently available [36,37,38]. The injection of iron and 6-hydroxydopamine has been shown to increase the echogenicity of the SN, providing further evidence for this mechanism [39]. It appears that pathological processes in the retina and the SN develop in parallel, driven by different mechanisms, but appear early in the course of the disease, even before the development of motor symptoms.

The potential interconnection of SN hyperechogenicity and retinal thickness is interesting, although they do not share the same pathological explanation. This specific correlation (r = −0.248), although mild, suggests that central macular thickness might be a promising biomarker candidate. While TCS findings are considered relatively unchangeable during follow-up [40], our own unpublished longitudinal data (presented in the Supplementary Materials) showed that while the GCIPL of both eyes and the outer macular segments of the left eye changed over 2.5 years, the central macula remained stable. This “stability” of the central macula mirrors the well-known stability of the TCS finding [40], suggesting they might both reflect early or structural markers of the disease. We did not find a correlation between SN area and RNFL thickness. Recently, Shi et al. [41] indicated that the mean RNFL thickness partly reflects striatal dopamine uptake, suggesting its value for early diagnosis. This mechanism is strongly reinforced by the findings of Ahn et al. (2018), who demonstrated that inner retinal thinning significantly correlates with the loss of nigral dopaminergic transporters on PET imaging, proving a direct in vivo cross-talk between macular architecture and central dopaminergic status [30].

While this study focuses on structural OCT, future neurodegenerative research should integrate both structural and vascular retinal imaging biomarkers through standardized multimodal approaches. To ensure reproducibility and clinical translation, subsequent studies must adhere to unified reporting frameworks and terminology. Specifically, future protocols should implement the international consensus recommendations for OCT angiography nomenclature and standardized imaging reporting established by Munk et al. (2021, 2022) [42,43]. Adopting these standardized frameworks will reduce reporting heterogeneity, establishing a more rigorous foundation for validating synergistic structural and vascular retinal biomarkers.

### Study Limitations

Although our study brings some interesting results, it has certain limitations that should be kept in mind when interpreting the results. First, our study employed a cross-sectional design, which measures both exposure and outcome at the same time; therefore, it does not allow for making causal inferences. Second, we were not able to analyze the influence of clinical subtypes of PD on SN echogenicity due to the small number of screened patients and the number of dropouts caused by non-transparent bone windows. The sample size is relatively modest, particularly after the exclusion of patients with inadequate temporal bone windows, which may reduce statistical power. Also, functional visual outcomes and their correlation with clinical disability were not explored, limiting the clinical applicability. Diagnostic sensitivity, specificity, and predictive performance were not evaluated. Finally, despite the presence of a correlation, it is mild, and subsequently, the clinical applicability at this stage is limited.

## 5. Conclusions

Our study indicates a mild cross-sectional association between SN hyperechogenicity and thinning of the central macula (M1) in patients with PD. The observed stability of the central macula over time mirrors the well-known characteristic temporal stability of SN hyperechogenicity. These findings suggest that the central macula might serve as a promising complementary biomarker candidate to TCS. The possible future clinical significance of these findings will have to be reanalyzed in further longitudinal studies with larger cohorts and intra-subject correlation analyses.

## Figures and Tables

**Table 1:** Retinal thickness values (GCIPL, average RNFL and macular segments thickness) in PD patients compared to HC.

Parameter (µm)	Patients with PD (n = 86 Patients, 172 Eyes)	Healthy Control Subjects (n = 86 Patients, 172 Eyes)	*p*
GCIPL	81.1 ± 7.2	85.4 ± 7.9	<0.001 **
Average RNFL	98.9 ± 10.0	106.1 ± 7.8	<0.001 **
Central macula, M1	267.7 ± 32.4	318.6 ± 16.6	<0.001 **
Inner superior segment, M2	311.7 ± 23.5	320.6 ± 12.4	<0.001 **
Inner nasal segment, M3	315.2 ± 26.7	318.4 ± 17.8	0.191
Inner inferior segment, M4	306.0 ± 22.1	310.5 ± 19.4	0.048 *
Inner temporal segment, M5	303.9 ± 24.8	319.0 ± 17.2	<0.001 **
Outer superior segment, M6	290.6 ± 23.6	326.7 ± 11.7	<0.001 **
Outer nasal segment, M7	290.5 ± 25.2	290.3 ± 35.7	0.958
Outer inferior segment, M8	285.5 ± 28.2	315.6 ± 15.2	<0.001 **
Outer temporal segment, M9	276.7 ± 26.9	289.9 ± 36.2	<0.001 **

Abbreviations: GCIPL: ganglion cell/inner plexiform layer; RNFL: retinal nerve fiber layer; M1–M9: macular segments determined by the Early Treatment Diabetic Retinopathy Study (ETDRS) grid, *—*p* < 0.05; **—*p* < 0.01.

**Table 2:** Retinal thickness values (GCIPL, average RNFL and macular segments thickness) in patients with normal or hyperechogenic SN.

Parameter (µm)	Pathological SN, >0.19	Normal SN, ≤0.19	*p*
GCIPL; n = 71 (142 eyes)	82.00 ± 7.483	79 36 ± 6.184	0.219
Average RNFL; n = 71 (142 eyes)	99.67 ± 9.731	100.75 ± 7.003	0.636
Central macula, M1; n = 71 (142 eyes)	263.47 ± 29.299	283.05 ± 27.944	0.006 **
Inner superior segment, M2; n = 71 (142 eyes)	310.96 ± 24.042	316.25 ± 17.447	0.347
Inner nasal segment, M3; n = 71 (142 eyes)	313.61 ± 26.549	316.55 ± 23.007	0.642
Inner inferior segment, M4; n = 71 (142 eyes)	304.17 ± 22.220	309.55 ± 18.726	0.308
Inner temporal segment, M5; n = 71 (142 eyes)	301.48 ± 23.146	311.55 ± 29.441	0.085
Outer superior segment, M6; n = 71 (142 eyes)	290.77 ± 23.869	297.05 ± 20.268	0.268
Outer nasal segment, M7; n = 71 (142 eyes)	293.35 ± 27.403	293.75 ± 24.294	0.951
Outer inferior segment, M8; n = 71 (142 eyes)	285.64 ± 26.385	287.25 ± 16.992	0.722
Outer temporal segment, M9; n = 71 (142 eyes)	275.39 ± 24.290	280.45 ± 24.313	0.389

Abbreviations: GCIPL: ganglion cell/inner plexiform layer; RNFL: retinal nerve fiber layer; M1–M9: macular segments determined by the Early Treatment Diabetic Retinopathy Study (ETDRS) grid; SN: substantia nigra; **—*p* < 0.01.

## Data Availability

The datasets generated and/or analyzed during the current study are available from the corresponding author upon reasonable request. These data are not publicly available because of ethical and privacy constraints designed to protect participant confidentiality.

## Supplementary Materials

The following supporting information can be downloaded at: https://www.mdpi.com/article/10.3390/biomedicines14071600/s1, Table S1: Retinal thickness values (GCIPL, average RNFL and macular segments thickness) in patients with PD in longitudinal study.

## Abbreviations

The following abbreviations are used in this manuscript: PD Parkinson’s disease, OCT Optical coherence tomography, SN Substantia nigra, TCS Transcranial parenchymal ultrasonography, RNFL Retinal nerve fiber layer, GCIPL Ganglion cell/inner plexiform layer, UCCS University Clinical Center of Serbia, HC Healthy controls, MDS Movement Disorder Society, MDS-UPDRS MDS-Unified Parkinson’s disease rating scale, ETDRS Early Treatment of Diabetic Retinopathy Study, SPSS Statistical Package for Social Sciences, M1 Central macula, M2 Inner superior segment, M3 Inner nasal segment, M4 Inner inferior segment, M5 Inner temporal segment, M6 Outer superior segment, M7 Outer nasal segment, M8 Outer inferior segment, M9 Outer temporal segment.
