# Supplementary material for: Association Between Substantia Nigra Hyperechogenicity and Central Macular Thickness in Parkinson’s Disease
Source: Biomedicines. 2026 Jul 17;14(7):1600. doi: 10.3390/biomedicines14071600 (PMC13406961; doi:10.3390/biomedicines14071600)
Supplement: Supplementary file 1 [file biomedicines-14-01600-s001.zip › biomedicines-4353385-supplementary.pdf]

Suplementary files

Table S1. Retinal thickness values (GCIPL, average RNFL and macular segments thickness) in patients with PD in longitudinal study.

	Changes of retinal thickness values Right eye	p	Changes of retinal thickness values Left eye	p
GCIPL	-16.6000 ± 6.4265	0.004**	-15.0000 ± 5.1478	0.003**
Average RNFL	2.8 ± 7.6	0.066	1.5 ± 4.0	0.059
**Central macula, M1**	3.2800 ± 29.8266	**0.588**	8.2800 ± 30.8997	**0.193**
Inner superior segment, M2	-1.0400 ± 29.6346	0.862	3.2000 ± 17.0758	0.358
Inner nasal segment, M3	-13.2000 ± 35.1082	0.072	-3.2400 ± 26.3491	0.544
Inner inferior segment, M4	-6.5200 ± 29.7911	0.285	-1.9600 ± 13.1513	0.463
Inner temporal segment, M5	-6.2400 ± 33.2745	0.358	-2.3200 ± 15.2608	0.455
Outer superior segment, M6	4.7200 ± 27.0671	0.392	8.7200 ± 19.1170	0.032*
Outer nasal segment, M7	2.2000 ± 27.3755	0.691	8.8400 ± 15.7762	0.010**
Outer inferior segment, M8	2.5200 ± 26.8718	0.643	8.9600 ± 16.2724	0.011*
Outer temporal segment, M9	-12.3200 ± 32.3364	0.069	-11.6000 ± 23.0742	0.019*

Abbreviations: GCIPL: ganglion cell/inner plexiform layer; RNFL: retinal nerve fiber layer; M1–M9: macular segments determined by the Early Treatment Diabetic Retinopathy Study (ETDRS) grid; * - $p < 0.05$; ** - $p < 0.01$.
